# Potential therapeutic value of miR‐425‐5p in metastatic colorectal cancer

**DOI:** 10.1111/jcmm.12902

**Published:** 2016-07-09

**Authors:** Ion Cristóbal, Juan Madoz‐Gúrpide, Federico Rojo, Jesús García‐Foncillas

**Affiliations:** ^1^ Translational Oncology DivisionOncohealth InstituteIIS‐Fundacion Jimenez Diaz‐UAMUniversity Hospital “Fundacion Jimenez Diaz”MadridSpain; ^2^ Pathology DepartmentIIS‐Fundacion Jimenez Diaz‐UAMUniversity Hospital “Fundacion Jimenez Diaz”MadridSpain

*Dear Editor:*

The recently published manuscript by Zhang *et al*. 1 provides novel important data about the role of miR‐425‐5p modulating chemosensitivity of colorectal cancer (CRC) cells to 5‐fluorouracil (5‐FU) and oxaliplatin treatments. The authors carried out a comparison between microRNA expression profiles from chemosensitive and chemoresistant HCT‐116 cells, and miR‐425‐5p was found markedly overexpressed in chemoresistant HCT‐116 cells. Interestingly, miR‐425‐5p inhibition had no effects on cell growth and apoptosis in untreated cells but restored chemosensitivity in HCT‐116 resistant cells both *in vitro* and *in vivo*.

These observations are of high therapeutic relevance since, as properly indicated by Zhang *et al*., 5‐FU and oxaliplatin are currently used as standard induction chemotherapy in metastatic CRC but, unfortunately, many patients develop chemoresistance during the disease progression. However, some issues should be taken into consideration. One criticism would be that those experiments showing enhanced chemosensitivity after miR‐425‐5p inhibition should have been done with 5‐FU in combination with oxaliplatin to mimic the FOLFOX regimen rather than separately. On the other hand, the development of HCT‐116 resistant cells is one key point in this study and obtaining up to 20‐fold increase in 5‐FU/oxaliplatin IC50 values is unusual when a resistant cell line is generated by a continuous exposure to these drugs. Thus, it would be very useful for reproducibility if the authors would have provided additional information at this regard such as how much time was needed to achieve this high chemoresistance. In concordance with this observation, it is also surprising that neither 5‐FU nor oxaliplatin were able to induce any effect in xenograft models injected with HCT‐116 resistant cells. One would expect that a combination of these agents could induce at least some modest tumour growth inhibition.

Furthermore, the authors demonstrated that PDCD10 is a novel direct target of miR‐425‐5p and claimed that the effects of miR‐425‐5p regulating chemosensitivity in CRC cells are dependent on PDCD10 modulation. However, it remains necessary to analyse the potential contribution of other described targets for this microRNA such as the tumour suppressor Phosphatase and tensin homologue (PTEN) 2. In fact, high PTEN expression has been reported to be associated with sensitivity to 5‐FU and oxaliplatin in CRC 3.

Although little is known about the functional significance of miR‐425‐5p, some data in the literature suggest that this microRNA could play a potential oncogenic role in cancer progression. As instance, it has been recently reported that miR‐425‐5p contributes to invasion and metastasis development in gastric cancer 4. We then investigated the status of miRNA‐425‐5p in CRC progression by Taqman low density arrays panel A (Applied Biosystems, Foster City, CA, USA) in 17 CRC patients with paired primary and metastatic tissue available. All samples were anonymized and de‐identified prior to analysis and the ethical committee and institutional review board approved the project. Relative gene expression analysis was performed with the 2^−ΔCt^ method and U6B as internal control. Interestingly, we found no differences in miR‐425‐5p between primary and metastatic tissues (fold change = 0.94; *P* = 0.842). These results would suggest that miR‐425‐5p deregulation occurs early in the primary tumour and its expression levels prior to metastatic progression may predict future response to chemotherapy. However, it seems not to be critical in the progression to metastatic disease. As a result of the short number of cases analysed confirmation in additional studies including larger cohorts is required.

In conclusion, the study by Zhang *et al*. provides novel important findings about the ability of miR‐425‐5p to regulate chemoresistance in CRC but the fact that their results were obtained only in HCT‐116 cells represents a major limitation of this study. Therefore, further investigation in additional CRC models with distinct PTEN status and PDCD10 expression are warranted to clarify the role of these two proteins in this issue and confirm miR‐425‐5p as a novel reliable therapeutic target in CRC. Moreover, it would be very interesting to assess in forthcoming studies the potential value of miR‐425‐5p deregulation as a predictive marker of response to FOLFOX regimen together with the contribution to chemoresistance of some other deregulated microRNAs identified by Zhang and colleagues in their work.

## Funding

This study was supported by PI13/02609 and PI15/00934 grants from ‘Instituto de Salud Carlos III FEDER’.

## Conflict of interest

The authors declare that they have no conflict of interest to disclose.
